# Distinct gene expression profiles in ovarian cancer linked to Lynch syndrome

**DOI:** 10.1007/s10689-014-9728-1

**Published:** 2014-05-22

**Authors:** Jenny-Maria Jönsson, Katarina Bartuma, Mev Dominguez-Valentin, Katja Harbst, Zohreh Ketabi, Susanne Malander, Mats Jönsson, Ana Carneiro, Anna Måsbäck, Göran Jönsson, Mef Nilbert

**Affiliations:** 1 Division of Oncology, Department of Clinical Sciences, Skane University Hospital, Lund University, 221 85 Lund, Sweden; 2 CREATE Health Strategic Center for Translational Cancer Research, Lund University, Lund, Sweden; 3 HNPCC-register, Clinical Research Centre, Hvidovre Hospital, Copenhagen University, Hvidovre, Denmark; 4 Department of Pathology, Institute of Laboratory Sciences, Skane University Hospital, Lund, Sweden

**Keywords:** HNPCC, Gene expression profiles, DASL, Candidate genes

## Abstract

Ovarian cancer linked to Lynch syndrome represents a rare subset that typically presents at young age as early-stage tumors with an overrepresentation of endometrioid and clear cell histologies. We investigated the molecular profiles of Lynch syndrome-associated and sporadic ovarian cancer with the aim to identify key discriminators and central tumorigenic mechanisms in hereditary ovarian cancer. Global gene expression profiling using whole-genome c-DNA-mediated Annealing, Selection, extension, and Ligation was applied to 48 histopathologically matched Lynch syndrome-associated and sporadic ovarian cancers. Lynch syndrome-associated and sporadic ovarian cancers differed by 349 significantly deregulated genes, including *PTPRH*, *BIRC3*, *SHH* and *TNFRSF6B.* The genes involved were predominantly linked to cell growth, proliferation, and cell-to-cell signaling and interaction. When stratified for histologic subtype, hierarchical clustering confirmed distinct differences related to heredity in the endometrioid and serous subtypes. Furthermore, separate clustering was achieved in an independent, publically available data set. The distinct genetic signatures in Lynch syndrome-associated and sporadic ovarian cancers point to alternative preferred tumorigenic routes and suggest that genetic discriminators may be relevant for molecular diagnostics and targeted therapeutics.

**Electronic supplementary material:**

The online version of this article (doi:10.1007/s10689-014-9728-1) contains supplementary material, which is available to authorized users.

## Introduction

Lynch syndrome is estimated to cause 2–4 % of ovarian cancer. Recognition of these cases is challenging, and many of the 9,000 ovarian cancers annually estimated to develop as part of Lynch syndrome probably escape detection. Whereas sporadic ovarian cancer and hereditary cancer caused by *BRCA1* and *BRCA2* gene mutations develop at a mean age of 65–70 years, typically show serous histopathology and present at advanced tumor stages [1, 2], ovarian cancer linked to Lynch syndrome typically develops at a mean age of 45 years as early-stage tumors of the endometrioid and clear cell histologic subtypes [2–7]. Lynch syndrome is caused by germline mutations in the mismatch-repair (MMR) genes *MLH1*, *MSH2*, *MSH6* and *PMS2.* Carriers of disease-predisposing mutations are estimated to be at 7–12 % life-time risk for ovarian cancer, at 50–80 % risk for colorectal cancer and at 40–60 % risk for endometrial cancer [5, 8, 9]. Recognition of ovarian cancers linked to Lynch syndrome tumors is important since family members at risk can be offered surveillance and/or prophylactic measures that reduce morbidity and mortality, not least from the more commonly occurring colorectal cancers.

In ovarian cancer, the different histopathologic subtypes have been suggested to constitute separate disease entities with differences related to biological features, treatment response and prognosis [10, 11]. A dualistic model for the development of ovarian cancer has been proposed. High-grade serous, high-grade endometrioid and undifferentiated carcinomas are thought to develop de novo, most likely from serous tubal intraepithelial carcinomas, whereas low-grade serous, low-grade endometrioid, mucinous and clear cell carcinomas show stepwise tumor development from precursors such as adenofibromas, borderline tumors and endometriosis [12, 13]. In line with this model, gene expression profiles differ between the various histologic subtypes as well as between invasive tumors and tumors of low-malignant potential [14, 15]. In colorectal cancer and in endometrial cancer, the MMR defective tumors are characterized by few gross genomic alterations and up-regulation of e.g. immune-regulatory genes. With the aim to identify gene expression profiles and genetic discriminators linked to MMR defective ovarian tumors, we applied global gene expression analysis to Lynch syndrome-associated and sporadic cancers.

## Materials and methods

### Tumor samples

We collected paraffin-embedded tumor tissue from Swedish and Danish Lynch syndrome mutation carriers and matched these tumors to sporadic ovarian cancers to correct for differences related to histopathology [15]. Histopathologic subtype and grade were determined according to Silverberg and to the WHO guidelines [16–18]. Hematoxylin & Eosin stained slides were reviewed by a gynecologic pathologist (AM) to verify histopathologic subtype and tumor grade. In total, 24 Lynch syndrome tumors from individuals with germline mutations in *MLH1* (n = 1), *MSH2* (n = 13) or *MSH6* (n = 10) and an associated loss of immunohistochemical MMR protein expression were included along with 24 sporadic ovarian cancers in which heredity had been excluded based on family history, normal MMR protein staining and normal results from *BRCA1* and *BRCA2* mutation analysis [1, 3, 19]. Clinical characteristics are outlined in Table 1 and detailed data are provided in online resource 1. Tumor tissue for immunohistochemical assessment of target genes was available from 46 tumors. Ethical approval for the study was granted from the ethics committee in Region Hovedstaden, Denmark and from the Lund University ethics committee, Sweden.

**Table 1:** Clinical characteristics of matched Lynch syndrome-associated and sporadic ovarian tumors in this study

	Lynch syndrome tumors	Sporadic tumors
	n = 24	n = 24
Age at diagnosis		
Median years (range)	47.0 (30–71)	57.0 (34–78)
Histologic subtype (%)		
Serous	10 (42)	10 (42)
Mucinous	0	0
Endometroid	7 (29)	7 (29)
Clear cell	7 (29)	7 (29)
Grade (%)		
1 (well)	9 (37.5)	11 (46)
2	10 (42)	6 (25)
3 (poor)	5 (20.5)	6 (25)
2/3	0	1 (4)
FIGO stage (%)		
I	13 (54)	11 (46)
II	2 (8.5)	5 (21)
III	4 (16.5)	7 (29)
IV	0	0
Unknown	5 (21)	1 (4)
Age of FFPE tissue		
Median years (range)	20.5 (3–54)	11.0 (10–28)

### RNA extraction and gene expression analysis

3–5 Tissue 10-μm sections were selected from non-necrotic tumor areas with >70 % tumor cell content. RNA was extracted using the High Pure RNA Paraffin Kit (Roche, Castle Hill, Australia) and RNA concentrations were determined using a NanoDrop Spectrophotometer (NanoDrop Technologies, Wilmington, DE) requiring 300 ng of RNA with 260/280 ratios >1.8. Gene expression analyses were performed at the SCIBLU Genomics Centre, Lund University, Sweden. The cDNA mediated Annealing, Selection, extension and Ligation (WG-DASL) assay (Illumina Inc, San Diego, CA) containing 24,526 probes, which represent 18,626 unique genes, was used for whole genome expression analysis. The samples were randomized on the chips and were profiled following the manufacturer’s instructions. BeadChips were then scanned on a BeadArray™ Reader using BeadScan software (v4.2), during which fluorescence intensities were read and images extracted.

### Data analysis

A raw average signal intensity >250 and >8,000 detected genes was required for further analysis of the samples. All 48 matched tumors met these criteria. The expression data were uploaded in the GenomeStudio software (Illumina Inc), quantile normalized and a presence filter of 80 % was applied to the probes across all samples with a detection *p* value of <0.01, leaving 12,897 probes for further analysis. The data were imported into MeV 4.6.02 software [20] and were log2 transformed and mean centered across assays. Unsupervised clustering using complete linkage hierarchical cluster analysis and Pearson correlation as similarity metric was performed. Two-class unpaired significance analysis of microarrays (SAM), including a permutation test using 100 permutations, was used to identify differentially expressed genes between the Lynch syndrome-associated and sporadic tumors at a false discovery rate (FDR) <0.01 [21]. Gene ontology analyses were generated through the use of Ingenuity Pathway Analysis (IPA; www.ingenuity.com). The data are available in NCBI’s Gene Expression Omnibus [22] through GEO Series accession number GSE37394. Technical reproducibility was granted through inclusion of duplicate samples, which demonstrated a mean correlation of 0.98 (range 0.90–0.99) and a mean r^2^ value of 0.96 (range 0.81–0.99). In order to ensure data robustness, data analysis was independently performed using alternative parameters and stricter criteria, i.e. cubic spline normalization and RefSeq features present in 70 % of the samples (*p* = 0.01). This approach left 3,380 probes that were further analyzed as described above (including cluster analyses, permutation test and leave-one-out test, followed by gene ontology analyses).

### Validation in an independent, publically available data set

The Lynch syndrome gene signature was validated using an independent, publically available data set consisting of 2,844 genes, mainly based on high-grade serous and endometrioid ovarian cancers [14]. The data were imported into MeV v4, log2 transformed and the probes were mean centred across assays. Unsupervised hierarchical clustering was performed as described above.

### Immunohistochemical staining

Immunohistochemical staining for key target genes was performed on fresh 3-µm sections from formalin-fixed, paraffin-embedded tumor tissue and the slides were mounted on ChemMate Capillary Gap Microscope Slides (DAKO A/S, Glostrup, Denmark). The sections were pretreated in PT Link (mTOR, PTEN) and Proteinase K (EGFR) according to the manufacturer’s instructions and stained in an automated immunostainer (TechMate 500 Plus, DAKO) with application of the DAKO EnVision™ Systems (DAKO) for visualization. The antibodies used included phosphorylated mTOR (p-mTOR, clone 49F, diluted 1:80, Cell Signaling Technology, Danvers, MA), PTEN (clone 6H2.1, diluted 1:100, DAKO) and EGFR (clone E30, diluted 1:25, DAKO). Evaluations were blinded to the hereditary status as well as to gene expression data and were performed independently by KB and JMJ. The p-mTOR stains were dichotomized as positive/negative, with any cytoplasmic p-mTOR staining considered positive. PTEN was evaluated as negative (no staining or weaker staining in the tumor cells compared to the surrounding tissues) or positive (equal or stronger cytoplasmic staining in tumor cells compared to surrounding tissues). EGFR staining was evaluated according to staining intensity (no, weak, moderate, or strong staining) and the percentage of stained tumor cells; tumors with >25 % of the cells moderately or intensely stained for EGFR were classified as positive [23–25]. MMR protein staining is outlined in Ketabi et al. [19].

### Statistical analysis

The Pearson correlation test was used to analyze gene expression data in duplicate samples. Fischer’s exact test was used to assess correlations between the immunohistochemical stainings. The analyses were conducted using the R software and SPSS software respectively (IBM SPSS version 19). *p* Values <0.05 were considered significant.

## Results

**Fig. 1:**
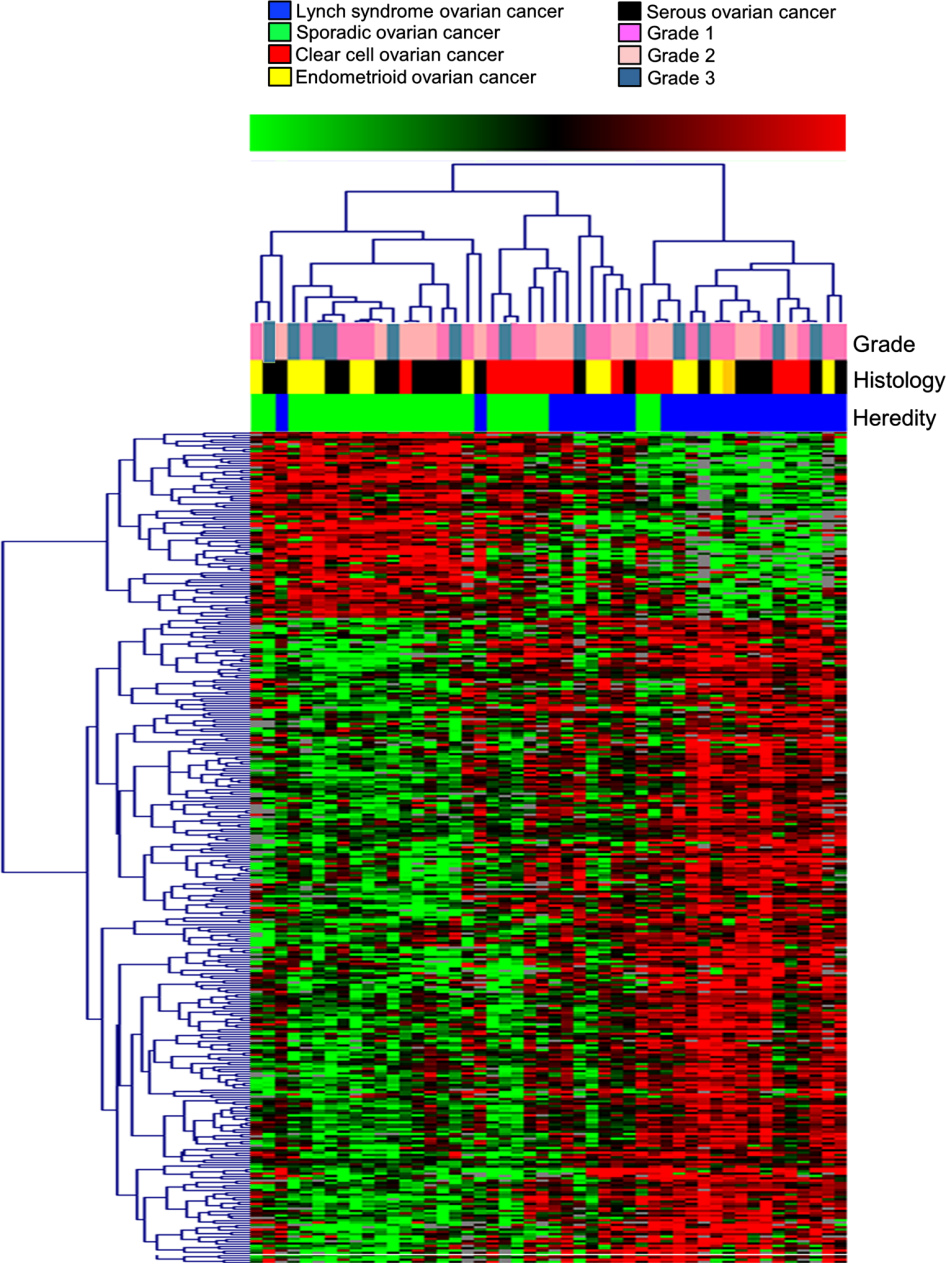
SAM analysis of differentially expressed genes (n = 349) in Lynch syndrome-associated and sporadic ovarian cancers at FDR < 0.01. Clustering was done using the TmeV application with the Pearson correlation distance metric for complete linkage

Unsupervised and supervised hierarchical cluster analysis in the matched dataset of 24 Lynch syndrome-associated and 24 sporadic tumors identified two major clusters related to hereditary status (online resource 2 and Fig. 1, respectively). SAM analysis identified 349 genes that were significantly deregulated between the Lynch syndrome tumors and the sporadic ovarian tumors (FDR < 0.01) (online resource 3). The top up-regulated genes in Lynch syndrome-associated tumors included e.g. *PTPRH*, *BIRC3*, *SHH* and *TNFRSF6B.* Enriched gene ontology processes were related to cellular growth and proliferation, cell death, and cell-to-cell signaling and interaction (Table 2). In sporadic ovarian cancers, SAM analysis identified up-regulation of e.g. *SHC1*, which is involved in protein tyrosine kinase activity, and *FSCN1*, which is related to protein binding (online resource 3).

**Table 2:** Enriched gene ontology processes in Lynch syndrome-associated ovarian cancer

Gene ontology processes	*p* value
Cell growth and proliferation	0.000010
Cell death and survival	0.000023
Cellular development	0.000050
Cellular function and maintenance	0.000050
Cell-to-cell signaling and interaction	0.000083

**Fig. 2:**
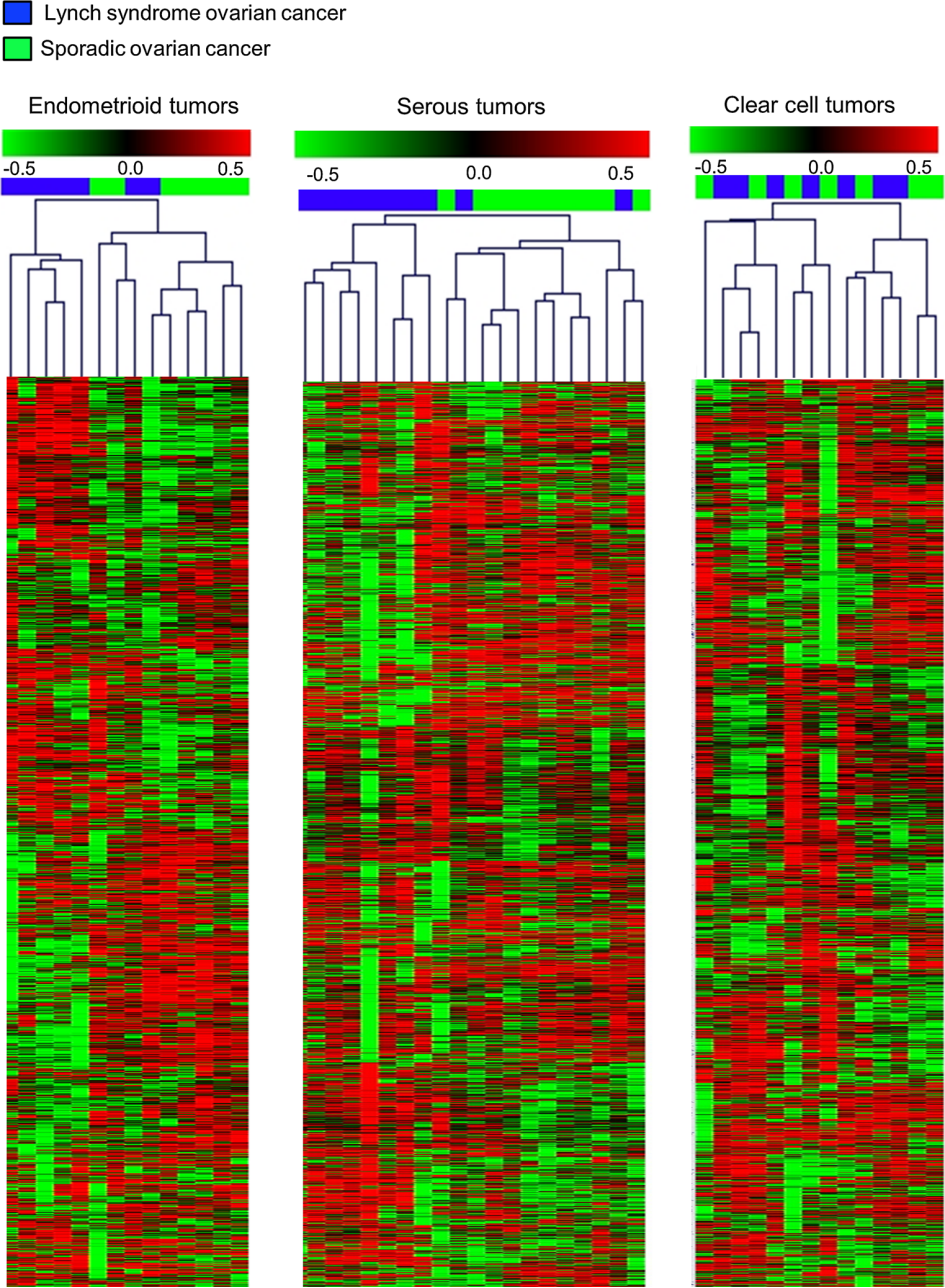
Unsupervised hierarchical cluster analysis of Lynch syndrome-associated and sporadic ovarian cancers in the separate histopathological subtypes. The analyses were performed using a 3,380 probe data set

Independent analysis using cubic spline normalization and requiring presence of RefSeq features in 70 % of the samples left 3,380 probes for analysis. Data stability was demonstrated using unsupervised hierarchical clustering, which resulted in identical clustering between Lynch syndrome tumors and sporadic tumors as in the original data set, and leave-one-out analysis, which correctly classified 79 % of the hereditary tumor samples and 62.5 % of the sporadic tumor samples. Based on these data, unsupervised hierarchical clustering was performed in the different histopathologic subtypes and identified clustering related to heredity in endometrioid and serous cancers, but not in clear cell cancers (Fig. 2). SAM analysis in the former subgroups identified 17 and 33 differentially expressed genes, respectively, between Lynch syndrome-associated and sporadic tumors (FDR < 0.01) (online resource 4).

**Fig. 3:**
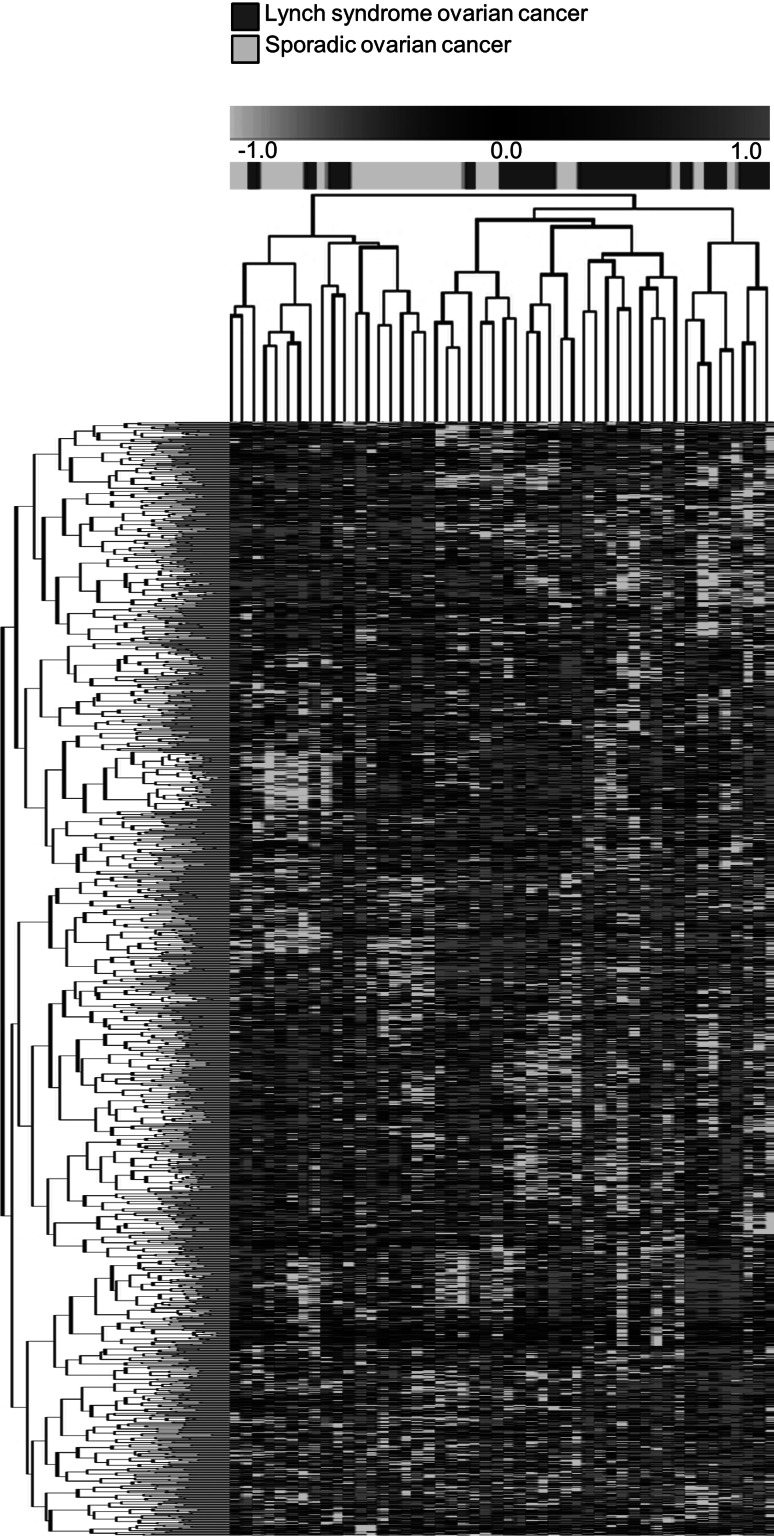
Unsupervised hierarchical cluster analysis based on 1,346 overlapping genes from an idependent, publically available dataset [14]. The Lynch syndrome-associated tumors cluster together

Application of a publically available 2,844 gene signature to our data identified 1,346 genes that were shared between the data sets [14]. Unsupervised hierarchical cluster analysis based on these 1,346 genes resulted in two main clusters with 20/24 Lynch syndrome tumors in one cluster, whereas the sporadic tumors were divided between the clusters (Fig. 3).

**Fig. 4:**
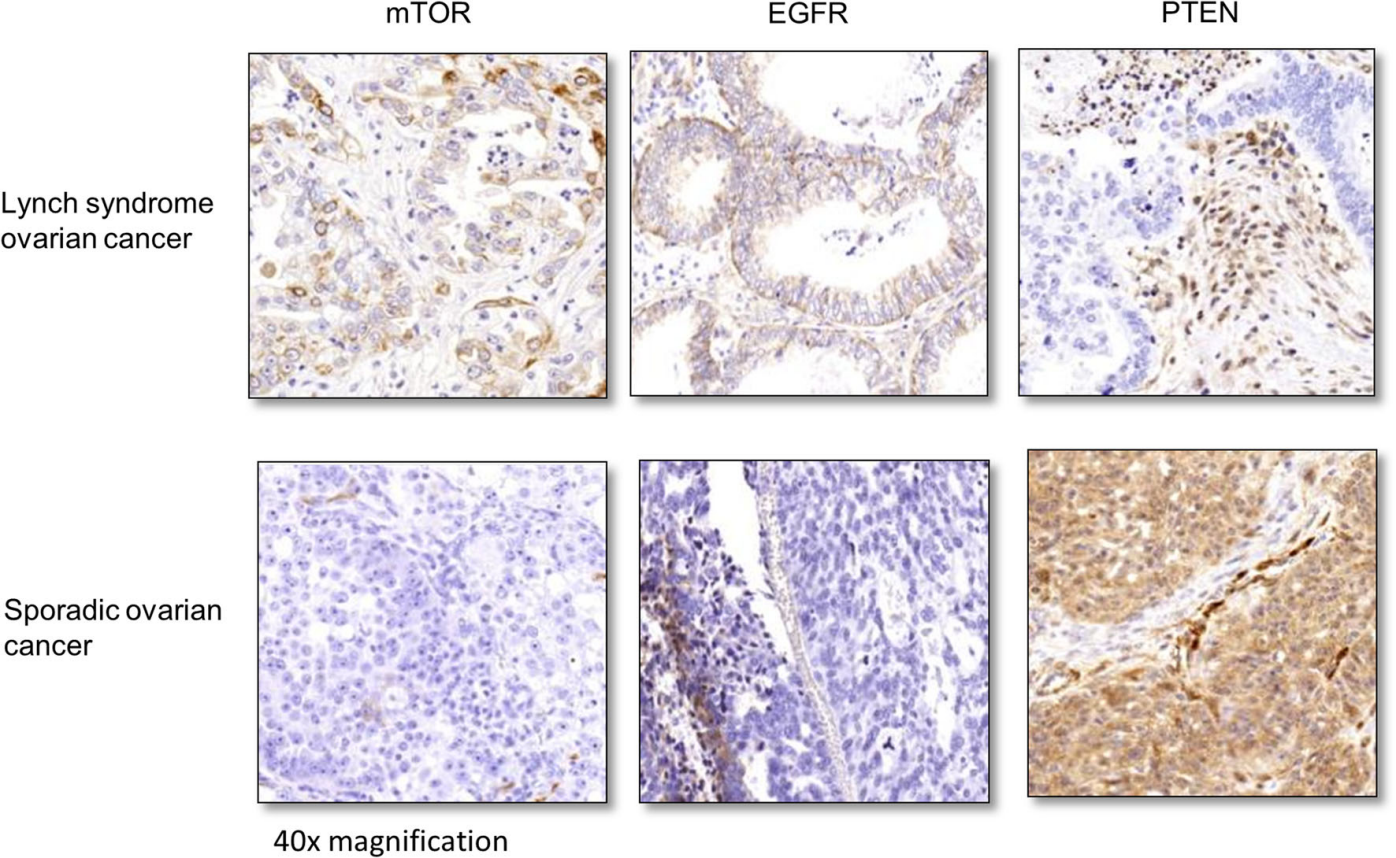
Immunohistochemical stainings for p-mTOR, EGFR and PTEN in ×40 magnification with Lynch syndrome-associated tumors presented on the top row and sporadic tumors on the bottom row. The left and middle columns show positive (*top row*) and negative (*bottom row*) p-mTOR and EGFR expression in tumor cells respectively. The right column shows negative PTEN expression in tumor cells but retained staining in surrounding tissue (*top row*) and positive PTEN expression in tumor cells and surrounding tissue (*bottom row*)

Immunohistochemical stainings demonstrated positivity for p-mTOR in 14/23 Lynch syndrome tumors and in 12/23 sporadic tumors (*p* = 0.767), positive staining for EGFR in 7/23 Lynch tumors and in 2/22 sporadic tumors (*p* = 0.135) and loss of PTEN in 17/23 Lynch syndrome tumors compared to in 14/23 sporadic tumors (*p* = 0.530) (Fig. 4).

## Discussion

Lynch syndrome represents a rare but distinctive cause of ovarian cancer. Knowledge about involved tumorigenic mechanisms, genotype-phenotype correlations and optimal treatment is limited and no data on gene expression profiles in Lynch syndrome-associated ovarian cancer are available. Whole-genome DASL-based gene expression profiling based on 18.6 k genes identified 349 significantly deregulated genes with up-regulation of e.g. *PTPRH*, *BIRC3*, *SHH* and *TNFRSF6B* in Lynch syndrome tumors. *PTPRH* is part of the protein tyrosine phosphatase family and has tumor suppressor as well as oncogenic functions. *BIRC3* has negative regulatory effect of the NFκβ signaling pathway, is associated with increased resistance to apoptosis and has also been linked to chemotherapy resistance [26, 27]. *SHH* is crucial in embryonic development and *TNFRSF6B* is a member of the tumor necrosis factor superfamily that mediates cell death. Sporadic ovarian cancers on the other hand show up-regulation of *SHC1*, that acts down-stream of *TP53* and is involved in cell migration and angiogenesis, and *FSCN1* that has been linked to invasive and metastatic potential in epithelial ovarian cancer [28]. Gene ontology analysis in Lynch syndrome tumors suggested involvement of genes related to cell growth, proliferation and cell death. The enrichment of cell growth and proliferation processes in Lynch syndrome ovarian cancers could potentially be linked to the predisposition for endometrioid tumors, which are typically low-grade tumors with low proliferation rates [13].

When the impact of heredity was analyzed within the different histopathologic subtypes, separate clustering was observed for endometrioid tumors and serous tumors (Fig. 2). These findings are based on small sample sets and need to be validated for further application. The lack of clustering within the clear cell cancer subset could potentially reflect a strong histology-related signature that overrules a potentially weaker hereditary signal, which is in line with distinct genetic alterations and clinical behavior in clear cell tumors [13, 29]. However, the finding of a stable genetic profile in Lynch syndrome-associated ovarian cancer is in line with previous studies on the impact from MMR deficiency for prognosis and prediction [4, 7, 8]. Clustering between Lynch syndrome tumors and sporadic tumors was achieved also when an independent, publically available data set was applied to our tumors (Fig. 3) [14]. In line with the observations by Tothill et al. [14] sub-clusters containing low-grade serous tumors and endometrioid tumors were identified, which may indicate distinct profiles also in these subtypes (data not shown).

The MAPK/ERK (MEK) signaling pathway is central in tumorigenesis and mutational activation has been suggested to have prognostic implications in ovarian cancer [30–34]. Mutations in *KRAS* and *BRAF*, which may activate the mTOR/PI3K/AKT pathway, are common in low-grade ovarian cancers (60 %) but rare in high-grade cancers [35]. Up-regulation of the mTOR pathway has been linked to poor prognosis, potentially through increased resistance to chemotherapeutic drugs such as paclitaxel and cisplatin in sporadic ovarian cancer [23, 36, 37]. Upregulation of both mTOR and MEK signaling has been demonstrated in Lynch syndrome-associated colorectal cancer, and Niskakoski et al. recently reported frequent mutations in *PIK3CA* and absence of *KRAS* and *BRAF* mutations in ovarian cancers linked to Lynch syndrome [38, 39]. Immunohistochemical staining for mTOR, EGFR and PTEN was motivated by these markers being key targets that have also been shown to be up-regulated in Lynch syndrome-associated colorectal cancer. Though frequent deregulation was observed, significant differences were not demonstrated, which could relate to other mechanisms of activation as well as alternative target proteins.

In summary, the gene expression profiles in Lynch syndrome-associated and sporadic ovarian cancers showed stable and reproducible differences with 349 significantly deregulated genes, which were primarily related to cellular growth, proliferation and cell death. Our findings point to differences in tumorigenesis and suggest that targets in the deregulated pathways may be relevant for diagnostic and therapeutic intervention in ovarian cancer linked to Lynch syndrome.

## Electronic supplementary material

Below is the link to the electronic supplementary material.

Supplementary material 1 (DOCX 22 kb)

Supplementary material 2 (TIFF 10463 kb)

Supplementary material 3 (TIFF 668 kb)

Supplementary material 4 (DOCX 42 kb)

Supplementary material 5 (DOCX 25 kb)
