# Supplementary material for: Distinct gene expression profiles in ovarian cancer linked to Lynch syndrome
Source: Fam Cancer. 2014 May 22;13(4):537–45. doi: 10.1007/s10689-014-9728-1 (PMC4231285; doi:10.1007/s10689-014-9728-1)
Supplement: Supplementary file 1 — Supplementary material 1 (DOCX 22 kb) [file 10689_2014_9728_MOESM1_ESM.docx]

**Online Resource 1**

**Title:** Distinct Gene Expression Profiles in Ovarian Cancer linked to Lynch Syndrome

**Journal:** Familial Cancer

**Authors:** Jenny-Maria Jönsson*, Katarina Bartuma*, Mev Dominguez-Valentin,

Katja Harbst, Zoreh Ketabi, Susanne Malander, Mats Jönsson, Ana Carneiro,

Anna Måsbäck, Göran Jönsson, Mef Nilbert

*These authors contributed equally

**Corresponding author:**

Jenny-Maria Jönsson

Division of Oncology, Department of Clinical Sciences, Lund University

221 85 Lund, Sweden

Telephone: +46-46-177860, Fax: +46-46-147327

E-mail: Jenny-Maria.Jonsson@med.lu.se

| Online Resource 1 | |  |  |  |  |  |  |  |
| --- | --- | --- | --- | --- | --- | --- | --- | --- |
| Lynch syndrome-associated and sporadic ovarian tumors | | | | | |  |  |  |
| matched for histology, grade, stage and age | | | | | |  |  |  |
| Lynch syndrome tumors | | | |  | Sporadic tumors | | | |
| Histological subtype | Grade | Stage | Age |  | Histological subtype | Grade | Stage | Age |
| Clear cell | 1 | IB | 42 |  | Clear cell | 1 | IC | 38 |
| Clear cell | 2 | IA | 49 |  | Clear cell | 1 | IC | 54 |
| Clear cell | 2 | IC | 54 |  | Clear cell | 1 | na | 51 |
| Clear cell | 2 | IB | 47 |  | Clear cell | 1 | IC | 60 |
| Clear cell | 2 | IC | 47 |  | Clear cell | 2 | IA | 34 |
| Clear cell | 2 | IIIC | 50 |  | Clear cell | 2 | IA | 40 |
| Clear cell | 3 | IC | 48 |  | Clear cell | 3 | N/A | 59 |
|  |  |  |  |  |  |  |  |  |
| Endometrioid | 1 | IA | 39 |  | Endometriod | 1 | IA | 56 |
| Endometrioid | 1 | IA | 39 |  | Endometrioid | 1 | IIB | 51 |
| Endometrioid | 1 | IC | 47 |  | Endometrioid | 1 | IIC | 76 |
| Endometrioid | 1 | IIA | 61 |  | Endometrioid | 1 | IIIC | 46 |
| Endometrioid | 1 | N/A | 40 |  | Endometrioid | 1 | IIIC | 60 |
| Endometrioid | 1 | IIIC | 50 |  | Endometrioid | 3 | IIC | 55 |
| Endometrioid | 3 | IC | 71 |  | Endometrioid | 3 | IIC | 78 |
|  |  |  |  |  |  |  |  |  |
| Serous | 1 | N/A | 48 |  | Serous | 1 | IA | 40 |
| Serous | 1 | IIIC | 47 |  | Serous | 1 | IIC | 64 |
| Serous | 2 | IC | 30 |  | Serous | 2 | IIIA | 62 |
| Serous | 2 | IIA | 40 |  | Serous | 2 | IIIB | 62 |
| Serous | 2 | N/A | 35 |  | Serous | 2 | IIIB | 64 |
| Serous | 2 | N/A | 64 |  | Serous | 2 | IIIC | 58 |
| Serous | 2 | IIIC | 67 |  | Serous | ´2/3 | IC | 62 |
| Serous | 3 | IA | 39 |  | Serous | 3 | IC | 51 |
| Serous | 3 | IA | 41 |  | Serous | 3 | IC | 61 |
| Serous | 3 | N/A | 54 |  | Serous | 3 | IIIC | 48 |
