# Supplementary figures and images for: Distinct gene expression profiles in ovarian cancer linked to Lynch syndrome
Source: Fam Cancer. 2014 May 22;13(4):537–45. doi: 10.1007/s10689-014-9728-1 (PMC4231285; doi:10.1007/s10689-014-9728-1)

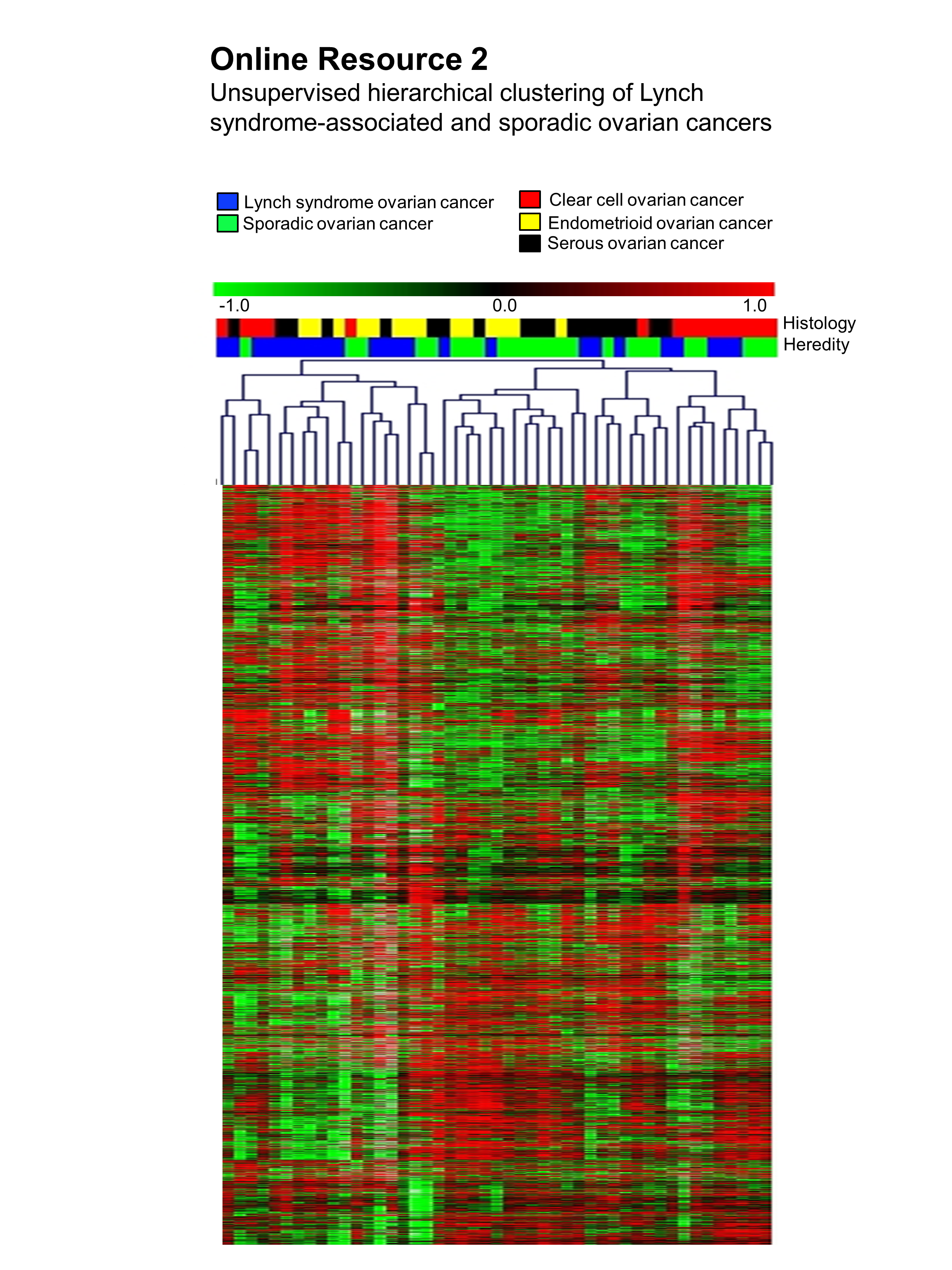

Supplement: Supplementary file 2 — Supplementary material 2 (TIFF 10463 kb) [file 10689_2014_9728_MOESM2_ESM.tif]

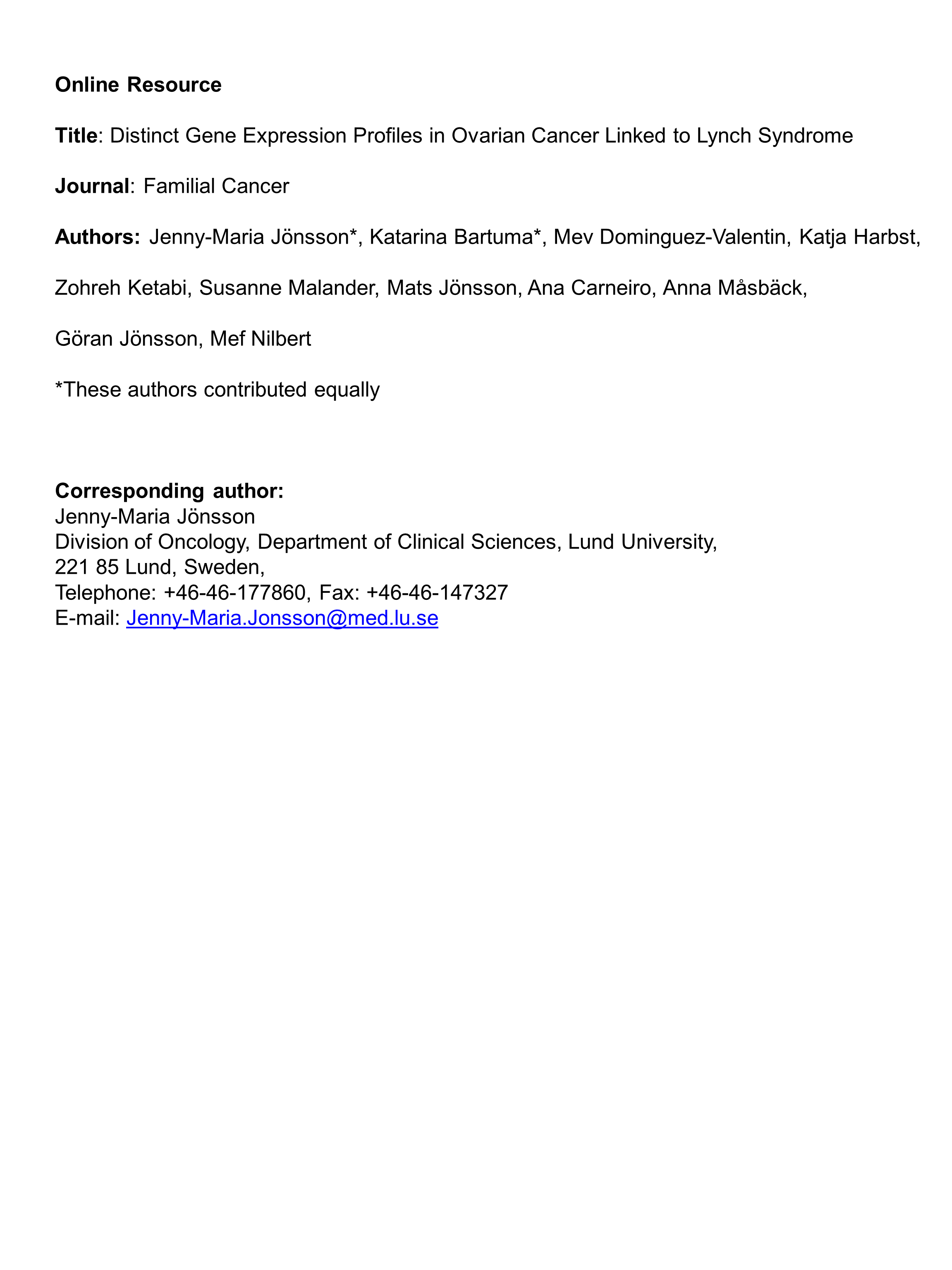

Supplement: Supplementary file 3 — Supplementary material 3 (TIFF 668 kb) [file 10689_2014_9728_MOESM3_ESM.tif]
