# Supplementary material for: Distinct gene expression profiles in ovarian cancer linked to Lynch syndrome
Source: Fam Cancer. 2014 May 22;13(4):537–45. doi: 10.1007/s10689-014-9728-1 (PMC4231285; doi:10.1007/s10689-014-9728-1)
Supplement: Supplementary file 4 — Supplementary material 4 (DOCX 42 kb) [file 10689_2014_9728_MOESM4_ESM.docx]

**Online Resource 3**

**Title:** Distinct Gene Expression Profiles in Ovarian Cancer linked to Lynch Syndrome

**Journal:** Familial Cancer

**Authors:** Jenny-Maria Jönsson*, Katarina Bartuma*, Mev Dominguez-Valentin,

Katja Harbst, Zoreh Ketabi, Susanne Malander, Mats Jönsson, Ana Carneiro,

Anna Måsbäck, Göran Jönsson, Mef Nilbert

*These authors contributed equally

**Corresponding author:**

Jenny-Maria Jönsson

Division of Oncology, Department of Clinical Sciences, Lund University

221 85 Lund, Sweden

Telephone: +46-46-177860, Fax: +46-46-147327

E-mail: Jenny-Maria.Jonsson@med.lu.se

| Online Resource 3 | | | |  | |  | |  | |  | |
| --- | --- | --- | --- | --- | --- | --- | --- | --- | --- | --- | --- |
| Genes (n=349) with differential expression at FDR <0.01 | | | | | | | | | |  | |
| All upregulated genes in Lynch syndrome-associated and | | | | | | | | | |  | |
| sporadic ovarian cancers respectively. | | | | | |  | |  | |  | |
|  | |  | |  | |  | |  | |  | |
| **Genes upregulated in Lynch syndrome-associated ovarian cancer** | | | | | | | | | | | |
| *Gene symbol* | | Fold change | | q-value (%) | | *Gene symbol* | | Fold change | | q-value (%) | |
| *TM4SF4* | | 2,874 | | 0,615 | | *TBL3* | | 1,828 | | 1,514 | |
| *TGM3* | | 2,688 | | 0,481 | | *NR1H2* | | 1,825 | | 0,000 | |
| *GLYCTK* | | 2,445 | | 0,000 | | *SEC24B* | | 1,825 | | 0,000 | |
| *ATAD4* | | 2,355 | | 1,275 | | *ARMC7* | | 1,815 | | 0,831 | |
| *PTPRH* | | 2,355 | | 0,000 | | *LIG1* | | 1,810 | | 1,155 | |
| *BIRC3* | | 2,345 | | 0,000 | | *HMBS* | | 1,809 | | 0,893 | |
| *LGALS7* | | 2,330 | | 0,719 | | *GJB3* | | 1,799 | | 1,450 | |
| *SHH* | | 2,316 | | 0,688 | | *MPST* | | 1,798 | | 0,740 | |
| *GPR35* | | 2,293 | | 0,000 | | *TOP2A* | | 1,791 | | 0,000 | |
| *CDCA7* | | 2,293 | | 0,000 | | *AGPAT2* | | 1,788 | | 0,640 | |
| *TNFRSF6B* | | 2,269 | | 1,230 | | *MKNK2* | | 1,786 | | 0,885 | |
| *TGIF1* | | 2,250 | | 0,000 | | *FLJ20125* | | 1,779 | | 1,155 | |
| *NR6A1* | | 2,232 | | 0,000 | | *NLE1* | | 1,770 | | 0,000 | |
| *TCF19* | | 2,130 | | 0,735 | | *MTP18* | | 1,766 | | 0,000 | |
| *CHDH* | | 2,114 | | 0,488 | | *NRF1* | | 1,756 | | 0,000 | |
| *SLC19A1* | | 2,094 | | 0,000 | | *TMEM145* | | 1,751 | | 0,000 | |
| *CD44* | | 2,076 | | 0,000 | | *RNFT1* | | 1,751 | | 0,831 | |
| *TRIM16* | | 2,043 | | 0,727 | | *C6orf26* | | 1,734 | | 1,077 | |
| *DLX5* | | 2,039 | | 0,000 | | *PHKB* | | 1,732 | | 1,074 | |
| *FAM111B* | | 2,025 | | 1,552 | | *HIST2H3C* | | 1,721 | | 0,615 | |
| *DHRSX* | | 2,000 | | 0,640 | | *LOC57228* | | 1,721 | | 0,000 | |
| *ACOT7* | | 1,999 | | 0,000 | | *DEADC1* | | 1,716 | | 1,155 | |
| *POU5F1* | | 1,977 | | 0,000 | | *PTK6* | | 1,715 | | 1,215 | |
| *C1orf95* | | 1,967 | | 0,000 | | *SLC37A3* | | 1,712 | | 0,000 | |
| *MST1R* | | 1,936 | | 0,831 | | *ADK* | | 1,712 | | 0,615 | |
| *EIF4EBP1* | | 1,936 | | 0,753 | | *CCDC134* | | 1,710 | | 0,000 | |
| *MOGAT1* | | 1,933 | | 1,077 | | *AIM1* | | 1,709 | | 0,688 | |
| *RGPD7* | | 1,916 | | 0,000 | | *ZNF417* | | 1,706 | | 0,674 | |
| *GALE* | | 1,914 | | 0,000 | | *HIST1H4A* | | 1,706 | | 1,341 | |
| *MAGI3* | | 1,913 | | 1,230 | | *LAMB3* | | 1,698 | | 1,155 | |
| *C11orf35* | | 1,890 | | 1,098 | | *ARFIP1* | | 1,689 | | 1,155 | |
| *NUPL1* | | 1,890 | | 0,000 | | *FLCN* | | 1,687 | | 1,361 | |
| *LOC201725* | | 1,880 | | 0,653 | | *METTL2A* | | 1,687 | | 0,000 | |
| *F3* | | 1,870 | | 1,488 | | *CXCL2* | | 1,683 | | 0,688 | |
| *TXNDC16* | | 1,859 | | 0,831 | | *SRCAP* | | 1,680 | | 1,155 | |
| *E2F8* | | 1,847 | | 1,155 | | *CSF2RB* | | 1,676 | | 1,084 | |
| *C16orf56* | | 1,837 | | 0,000 | | *PLAUR* | | 1,676 | | 0,615 | |
| *CCDC150* | | 1,837 | | 0,372 | | *DOT1L* | | 1,675 | | 1,077 | |
| *TMEM194* | | 1,835 | | 0,000 | | *AP1B1* | | 1,672 | | 0,893 | |
| **Genes upregulated in Lynch syndrome-associated ovarian cancer** | | | | | | | | | | | |
| *Gene symbol* | | Fold change | | q-value (%) | | *Gene symbol* | | Fold change | | q-value (%) | |
| *ACOX1* | | 1,667 | | 0,000 | | *EXOC6* | | 1,560 | | 0,000 | |
| *TMEM37* | | 1,665 | | 1,155 | | *KENAE* | | 1,558 | | 1,488 | |
| *DNAJB2* | | 1,665 | | 1,155 | | *IER3* | | 1,555 | | 0,000 | |
| *LLGL2* | | 1,664 | | 1,084 | | *ARMC8* | | 1,550 | | 1,378 | |
| *MLX* | | 1,661 | | 0,688 | | *IKZF3* | | 1,548 | | 0,893 | |
| *PIF1* | | 1,661 | | 0,615 | | *EPHA2* | | 1,547 | | 0,615 | |
| *PRELID2* | | 1,661 | | 1,155 | | *C18orf19* | | 1,546 | | 0,000 | |
| *DNASE1L1* | | 1,659 | | 0,831 | | *CCDC97* | | 1,539 | | 0,674 | |
| *LIN7B* | | 1,658 | | 1,155 | | *HIST1H3H* | | 1,533 | | 0,000 | |
| *KITLG* | | 1,657 | | 0,615 | | *CHRNA10* | | 1,529 | | 0,893 | |
| *SLC9A2* | | 1,656 | | 1,155 | | *LOC440350* | | 1,524 | | 1,077 | |
| *VEGFA* | | 1,656 | | 1,077 | | *CGI-96* | | 1,524 | | 1,155 | |
| *STBD1* | | 1,654 | | 0,615 | | *SIGIRR* | | 1,520 | | 1,378 | |
| *ITGA2* | | 1,650 | | 0,000 | | *KRT19* | | 1,519 | | 1,155 | |
| *TNIP2* | | 1,636 | | 0,000 | | *BAIAP2L2* | | 1,518 | | 1,246 | |
| *VPS25* | | 1,636 | | 0,000 | | *NUTF2* | | 1,514 | | 0,615 | |
| *TSPAN13* | | 1,635 | | 0,615 | | *SMARCAD1* | | 1,514 | | 1,488 | |
| *CLN6* | | 1,632 | | 0,000 | | *NCAPH2* | | 1,511 | | 1,488 | |
| *CDCA2* | | 1,632 | | 1,077 | | *LOC653604* | | 1,510 | | 0,719 | |
| *SGTA* | | 1,628 | | 1,514 | | *WDHD1* | | 1,510 | | 1,077 | |
| *ABCC1* | | 1,628 | | 0,893 | | *UFD1L* | | 1,509 | | 1,155 | |
| *C19orf48* | | 1,626 | | 0,000 | | *PIH1D1* | | 1,507 | | 0,000 | |
| *SREBF1* | | 1,624 | | 1,246 | | *NT5DC3* | | 1,506 | | 1,155 | |
| *TSPO* | | 1,621 | | 1,337 | | *CENPK* | | 1,505 | | 0,831 | |
| *BTBD3* | | 1,620 | | 0,893 | | *CTDP1* | | 1,504 | | 1,155 | |
| *PFKFB4* | | 1,614 | | 0,000 | | *PUS1* | | 1,502 | | 0,000 | |
| *KIF13B* | | 1,609 | | 1,155 | | *CALML4* | | 1,502 | | 1,246 | |
| *TBCE* | | 1,608 | | 0,615 | | *LARP2* | | 1,501 | | 0,000 | |
| *FANCA* | | 1,603 | | 0,715 | | *CHMP7* | | 1,500 | | 0,893 | |
| *CDCA3* | | 1,601 | | 0,831 | | *RAB15* | | 1,498 | | 1,077 | |
| *SDCCAG3* | | 1,598 | | 0,615 | | *RAB24* | | 1,497 | | 0,000 | |
| *KIAA1543* | | 1,596 | | 1,055 | | *C14orf142* | | 1,495 | | 1,077 | |
| *HIST1H2AK* | | 1,590 | | 0,496 | | *GGA1* | | 1,493 | | 1,077 | |
| *HSF4* | | 1,588 | | 1,077 | | *CYB5R4* | | 1,492 | | 1,155 | |
| *PSMC3IP* | | 1,586 | | 0,615 | | *ADCY6* | | 1,491 | | 0,831 | |
| *ATP5G1* | | 1,578 | | 1,378 | | *GPHN* | | 1,490 | | 1,155 | |
| *LAD1* | | 1,577 | | 0,000 | | *KIF23* | | 1,489 | | 0,615 | |
| *PHKG2* | | 1,574 | | 0,753 | | *TATDN2* | | 1,489 | | 0,615 | |
| *REPIN1* | | 1,572 | | 0,615 | | *LONP1* | | 1,488 | | 0,000 | |
| *MAMDC4* | | 1,572 | | 0,000 | | *TRAF4* | | 1,487 | | 0,000 | |
| *BBC3* | | 1,569 | | 0,615 | | *LOC641522* | | 1,487 | | 1,230 | |
| *TNFRSF10B* | | 1,565 | | 1,155 | | *KIAA1919* | | 1,485 | | 1,378 | |
| *HIP1R* | | 1,565 | | 1,155 | | *C13orf3* | | 1,483 | | 1,552 | |
| *POMT1* | | 1,564 | | 1,378 | | *HIF1A* | | 1,483 | | 0,805 | |
| **Genes upregulated in Lynch syndrome-associated ovarian cancer** | | | | | | | | | | | |
| *Gene symbol* | | Fold change | | q-value (%) | | *Gene symbol* | | Fold change | | q-value (%) | |
| *CLK3* | | 1,483 | | 1,147 | | *MCMDC1* | | 1,380 | | 0,000 | |
| *PPP1R12C* | | 1,481 | | 0,481 | | *TCOF1* | | 1,376 | | 0,831 | |
| *METAP1* | | 1,470 | | 0,000 | | *WDR82* | | 1,374 | | 0,000 | |
| *TRIM13* | | 1,468 | | 0,000 | | *CRK* | | 1,374 | | 1,021 | |
| *ADAM9* | | 1,467 | | 0,500 | | *TKT* | | 1,373 | | 1,155 | |
| *THAP6* | | 1,465 | | 0,893 | | *PIK3R1* | | 1,369 | | 0,500 | |
| *COQ9* | | 1,463 | | 0,893 | | *ANAPC2* | | 1,369 | | 1,514 | |
| *FUT10* | | 1,462 | | 0,000 | | *U2AF2* | | 1,367 | | 0,893 | |
| *RERE* | | 1,453 | | 1,275 | | *QPCTL* | | 1,367 | | 1,155 | |
| *NSF* | | 1,446 | | 0,000 | | *TADA2L* | | 1,366 | | 0,831 | |
| *LSM7* | | 1,446 | | 0,000 | | *ATF4* | | 1,364 | | 0,893 | |
| *CISD2* | | 1,445 | | 0,719 | | *LIPA* | | 1,362 | | 1,378 | |
| *DEF8* | | 1,444 | | 0,893 | | *C2orf24* | | 1,361 | | 1,275 | |
| *PDZD8* | | 1,444 | | 0,831 | | *ST14* | | 1,355 | | 0,615 | |
| *BTBD7* | | 1,443 | | 1,084 | | *ZCCHC17* | | 1,354 | | 0,000 | |
| *COBRA1* | | 1,438 | | 0,753 | | *VARS* | | 1,353 | | 0,965 | |
| *PPP1R13L* | | 1,436 | | 1,155 | | *SELI* | | 1,352 | | 1,378 | |
| *BCR* | | 1,434 | | 1,155 | | *ZMIZ2* | | 1,346 | | 1,361 | |
| *CCNA2* | | 1,434 | | 0,893 | | *SBNO1* | | 1,344 | | 0,831 | |
| *HIST1H2AI* | | 1,432 | | 1,147 | | *B4GALT7* | | 1,341 | | 1,077 | |
| *ADNP2* | | 1,431 | | 0,500 | | *RBM23* | | 1,335 | | 0,753 | |
| *VPS13A* | | 1,427 | | 0,000 | | *PPAT* | | 1,328 | | 0,615 | |
| *TMEM1* | | 1,426 | | 0,000 | | *PRMT1* | | 1,321 | | 1,155 | |
| *SEC31B* | | 1,426 | | 1,155 | | *NIN* | | 1,320 | | 0,000 | |
| *C18orf22* | | 1,423 | | 0,000 | | *FTL* | | 1,320 | | 0,753 | |
| *RPL6* | | 1,414 | | 0,000 | | *RAC1* | | 1,319 | | 1,246 | |
| *GMEB1* | | 1,414 | | 0,615 | | *TMEM199* | | 1,319 | | 1,246 | |
| *ADAT1* | | 1,413 | | 0,000 | | *ZDHHC3* | | 1,318 | | 1,552 | |
| *dJ222E13.2* | | 1,413 | | 0,805 | | *SRP68* | | 1,315 | | 1,077 | |
| *DIP2A* | | 1,411 | | 1,341 | | *TXNDC5* | | 1,312 | | 0,753 | |
| *RPL34* | | 1,404 | | 0,000 | | *ICT1* | | 1,309 | | 1,155 | |
| *CDC42* | | 1,403 | | 1,552 | | *C19orf24* | | 1,309 | | 1,155 | |
| *HPS1* | | 1,402 | | 1,488 | | *GALK2* | | 1,306 | | 1,341 | |
| *ARL4A* | | 1,402 | | 0,516 | | *ZNF410* | | 1,306 | | 0,719 | |
| *KIAA1542* | | 1,399 | | 0,000 | | *ZBED4* | | 1,303 | | 0,000 | |
| *DSN1* | | 1,398 | | 1,077 | | *XPO5* | | 1,299 | | 0,653 | |
| *BACH1* | | 1,398 | | 1,246 | | *EFTUD2* | | 1,297 | | 0,000 | |
| *GRPEL1* | | 1,396 | | 1,155 | | *AKR1A1* | | 1,292 | | 1,077 | |
| *DSG2* | | 1,395 | | 1,155 | | *SDCCAG1* | | 1,290 | | 1,155 | |
| *G0S2* | | 1,391 | | 1,155 | | *KIAA0586* | | 1,287 | | 1,230 | |
| *UHRF1* | | 1,385 | | 1,055 | | *MTHFD1* | | 1,283 | | 0,615 | |
| *GINS3* | | 1,384 | | 1,488 | | *CS* | | 1,272 | | 1,246 | |
| *CBX8* | | 1,383 | | 1,155 | | *CDC123* | | 1,256 | | 1,155 | |
| *RTN3* | | 1,381 | | 0,893 | | *EIF4E2* | | 1,253 | | 1,378 | |
| **Genes upregulated in Lynch syndrome-associated ovarian cancer** | | | | | | | | | | | |
| *Gene symbol* | | Fold change | | q-value (%) | | *Gene symbol* | | Fold change | | q-value (%) | |
| *SNRP70* | | 1,251 | | 1,155 | |  | |  | |  | |
| *LOC91431* | | 1,249 | | 1,155 | |  | |  | |  | |
| *NME1* | | 1,249 | | 0,831 | |  | |  | |  | |
| *MRPL27* | | 1,225 | | 1,077 | |  | |  | |  | |
| *TRPM4* | | 1,213 | | 0,615 | |  | |  | |  | |
| *RPL17* | | 1,211 | | 0,885 | |  | |  | |  | |
| *RREB1* | | 1,190 | | 1,077 | |  | |  | |  | |
| *FBXW7* | | 1,146 | | 1,077 | |  | |  | |  | |
|  | |  | |  | |  | |  | |  | |
|  | |  | |  | |  | |  | |  | |
|  | |  | |  | |  | |  | |  | |
|  | |  | |  | |  | |  | |  | |
|  | |  | |  | |  | |  | |  | |
|  | |  | |  | |  | |  | |  | |
|  | |  | |  | |  | |  | |  | |
|  | |  | |  | |  | |  | |  | |
|  | |  | |  | |  | |  | |  | |
|  | |  | |  | |  | |  | |  | |
|  | |  | |  | |  | |  | |  | |
|  | |  | |  | |  | |  | |  | |
|  | |  | |  | |  | |  | |  | |
|  | |  | |  | |  | |  | |  | |
|  | |  | |  | |  | |  | |  | |
|  | |  | |  | |  | |  | |  | |
|  | |  | |  | |  | |  | |  | |
|  | |  | |  | |  | |  | |  | |
|  | |  | |  | |  | |  | |  | |
|  | |  | |  | |  | |  | |  | |
|  | |  | |  | |  | |  | |  | |
|  | |  | |  | |  | |  | |  | |
|  | |  | |  | |  | |  | |  | |
|  | |  | |  | |  | |  | |  | |
|  | |  | |  | |  | |  | |  | |
|  | |  | |  | |  | |  | |  | |
|  | |  | |  | |  | |  | |  | |
|  | |  | |  | |  | |  | |  | |
|  | |  | |  | |  | |  | |  | |
|  | |  | |  | |  | |  | |  | |
|  | |  | |  | |  | |  | |  | |
|  | |  | |  | |  | |  | |  | |
|  | |  | |  | |  | |  | |  | |
|  | |  | |  | |  | |  | |  | |
|  | |  | |  | |  | |  | |  | |
|  | |  | |  | |  | |  | |  | |
| **Genes upregulated in sporadic ovarian cancer** | | | | | | |  | |  | |  |
| Gene symbol | Fold change | | q-value (%) | | Gene symbol | | Fold change | | q-value (%) | |  |
| *SHC1* | 0,845 | | 1,454 | | *RGL1* | | 0,589 | | 0,520 | |  |
| *FSCN1* | 0,764 | | 0,995 | | *FLJ36070* | | 0,588 | | 0,955 | |  |
| *ITGB5* | 0,758 | | 0,000 | | *S100A13* | | 0,587 | | 1,454 | |  |
| *MYL9* | 0,756 | | 1,215 | | *RECK* | | 0,585 | | 0,520 | |  |
| *ASS1* | 0,706 | | 1,402 | | *PFKM* | | 0,583 | | 1,402 | |  |
| *ZNF323* | 0,704 | | 0,785 | | *BLCAP* | | 0,580 | | 0,785 | |  |
| *TRIM8* | 0,697 | | 1,454 | | *NFIX* | | 0,579 | | 0,000 | |  |
| *PRAF2* | 0,685 | | 0,520 | | *CLSTN2* | | 0,574 | | 1,021 | |  |
| *C1S* | 0,683 | | 1,361 | | *DHRS3* | | 0,574 | | 1,402 | |  |
| *GFOD1* | 0,680 | | 1,454 | | *KIAA0672* | | 0,569 | | 1,454 | |  |
| *C3orf1* | 0,678 | | 0,520 | | *SSPN* | | 0,568 | | 0,000 | |  |
| *FBXO32* | 0,677 | | 0,955 | | *FBXL13* | | 0,567 | | 0,955 | |  |
| *TCEAL3* | 0,666 | | 1,021 | | *MAP6* | | 0,560 | | 0,464 | |  |
| *C6orf204* | 0,665 | | 0,955 | | *SLC40A1* | | 0,560 | | 1,402 | |  |
| *KCTD10* | 0,665 | | 0,520 | | *SYN1* | | 0,557 | | 0,955 | |  |
| *OLFML1* | 0,662 | | 1,341 | | *KCNN3* | | 0,551 | | 0,955 | |  |
| *IL13RA1* | 0,662 | | 0,955 | | *ROBO3* | | 0,548 | | 0,520 | |  |
| *LTBP3* | 0,656 | | 0,744 | | *DKK3* | | 0,546 | | 0,995 | |  |
| *ABCA3* | 0,655 | | 1,215 | | *CFH* | | 0,545 | | 1,021 | |  |
| *SPARCL1* | 0,647 | | 1,402 | | *C1orf175* | | 0,542 | | 0,000 | |  |
| *OPTN* | 0,646 | | 1,215 | | *LARP6* | | 0,530 | | 0,748 | |  |
| *SLC41A3* | 0,641 | | 1,402 | | *MXRA5* | | 0,516 | | 0,000 | |  |
| *SARM1* | 0,636 | | 1,361 | | *SCARA3* | | 0,515 | | 0,520 | |  |
| *DPYSL3* | 0,636 | | 0,520 | | *KCNK15* | | 0,512 | | 0,520 | |  |
| *C1R* | 0,633 | | 0,520 | | *TSHZ3* | | 0,505 | | 0,000 | |  |
| *SLC6A9* | 0,629 | | 1,402 | | *NAALADL1* | | 0,504 | | 0,520 | |  |
| *PARP16* | 0,629 | | 0,520 | | *DZIP1* | | 0,504 | | 1,021 | |  |
| *LBH* | 0,627 | | 0,464 | | *TIAM1* | | 0,503 | | 0,000 | |  |
| *PKNOX2* | 0,625 | | 0,955 | | *SMARCD3* | | 0,500 | | 0,735 | |  |
| *KIAA1217* | 0,624 | | 0,995 | | *TPPP3* | | 0,496 | | 1,454 | |  |
| *BACE1* | 0,624 | | 0,520 | | *FNDC1* | | 0,496 | | 0,520 | |  |
| *PLXDC2* | 0,622 | | 0,000 | | *C20orf103* | | 0,488 | | 0,520 | |  |
| *ANGPTL2* | 0,616 | | 0,464 | | *BEXL1* | | 0,487 | | 1,454 | |  |
| *BST2* | 0,615 | | 0,805 | | *IFIT1* | | 0,486 | | 0,520 | |  |
| *CGNL1* | 0,613 | | 0,785 | | *PDE1A* | | 0,484 | | 0,464 | |  |
| *CTSF* | 0,612 | | 0,520 | | *RERG* | | 0,480 | | 0,000 | |  |
| *NRXN2* | 0,610 | | 1,402 | | *TFAP2C* | | 0,471 | | 1,454 | |  |
| *C5orf4* | 0,608 | | 1,021 | | *TGFBR3* | | 0,461 | | 0,520 | |  |
| *GAS6* | 0,607 | | 1,402 | | *COL13A1* | | 0,460 | | 0,995 | |  |
| *BCAM* | 0,602 | | 0,464 | | *TCEAL2* | | 0,440 | | 1,402 | |  |
| *FMO4* | 0,595 | | 1,402 | | *EPHB6* | | 0,419 | | 0,785 | |  |
| *EXT1* | 0,593 | | 1,402 | | *WIT-1* | | 0,358 | | 0,000 | |  |
| *NDN* | 0,591 | | 0,995 | | *WT1* | | 0,358 | | 0,000 | |  |
| *SPG20* | 0,589 | | 1,341 | |  | |  | |  | |  |
