# Supplementary material for: Distinct gene expression profiles in ovarian cancer linked to Lynch syndrome
Source: Fam Cancer. 2014 May 22;13(4):537–45. doi: 10.1007/s10689-014-9728-1 (PMC4231285; doi:10.1007/s10689-014-9728-1)
Supplement: Supplementary file 5 — Supplementary material 5 (DOCX 25 kb) [file 10689_2014_9728_MOESM5_ESM.docx]

**Online Resource 4**

**Title:** Distinct Gene Expression Profiles in Ovarian Cancer linked to Lynch Syndrome

**Journal:** Familial Cancer

**Authors:** Jenny-Maria Jönsson*, Katarina Bartuma*, Mev Dominguez-Valentin,

Katja Harbst, Zoreh Ketabi, Susanne Malander, Mats Jönsson, Ana Carneiro,

Anna Måsbäck, Göran Jönsson, Mef Nilbert

*These authors contributed equally

**Corresponding author:**

Jenny-Maria Jönsson

Division of Oncology, Department of Clinical Sciences, Lund University

221 85 Lund, Sweden

Telephone: +46-46-177860, Fax: +46-46-147327

E-mail: Jenny-Maria.Jonsson@med.lu.se

| Online Resource 4 | |  |  |  |  |
| --- | --- | --- | --- | --- | --- |
| All upregulated genes in Lynch syndrome-associated and | | | | |  |
| sporadic ovarian cancers in the respective histological subtypes*  at FDR<0.01 | | | | |  |
|  |  |  |  |  |  |
| **Genes upregulated in Lynch syndrome-associated endometrioid cancers** | | | | | |
| *Gene symbol* | Fold change | q-value (%) | *Gene symbol* | Fold change | q-value (%) |
| *ADCY6* | 2.1179445 | 0.0 | *PHF5A* | 1.6051805 | 0.0 |
| *GNL3* | 1.891987 | 0.0 | *ADSSL1* | 1.5837971 | 0.0 |
| *ATF4* | 1.7959982 | 0.0 | *FAM110A* | 1.454949 | 0.0 |
| *ZMIZ2* | 1.6508219 | 0.0 |  |  |  |
|  |  |  |  |  |  |
| **Genes upregulated in sporadic endometrioid cancers** | | | | |  |
| *Gene symbol* | Fold change | q-value (%) | *Gene symbol* | Fold change | q-value (%) |
| *PSAP* | 0.85969657 | 0.0 | *PRDX6* | 0.5816085 | 0.0 |
| *IFI6* | 0.79030377 | 0.0 | *TRIM8* | 0.57919014 | 0.0 |
| *TCEAL4* | 0.7092731 | 0.0 | *TMEM59* | 0.5730983 | 0.0 |
| *TUBA1B* | 0.6355207 | 0.0 | *UBE2E2* | 0.5562362 | 0.0 |
| *UHMK1* | 0.60661423 | 0.0 | *ADCY3* | 0.5295252 | 0.0 |
|  |  |  |  |  |  |
| **Genes upregulated in Lynch syndrome-associated serous cancers** | | | | | |
| *Gene symbol* | Fold change | q-value (%) | *Gene symbol* | Fold change | q-value (%) |
| *KITLG* | 2.5948038 | 0.0 | *UHRF1* | 1.5584956 | 0.0 |
| *TOP2A* | 2.0770216 | 0.0 | *EFTUD2* | 1.547197 | 0.0 |
| *PUS1* | 1.793897 | 0.0 | *FBXO9* | 1.5010511 | 0.0 |
| *CCNA2* | 1.7887809 | 0.0 | *LSM11* | 1.4372725 | 0.0 |
| *RPL6* | 1.7273504 | 0.0 | *MTHFD1* | 1.4250932 | 0.0 |
| *C18orf22* | 1.7147766 | 0.0 | *NOC2L* | 1.3192544 | 0.0 |
| *RPL34* | 1.6572405 | 0.0 | *BTBD6* | 1.3045322 | 0.0 |
| *ADNP2* | 1.6516519 | 0.0 | *TCEA1* | 1.2828215 | 0.0 |
| *WDR82* | 1.5924278 | 0.0 | *TRIM3* | 1.2612115 | 0.0 |
| *PRMT1* | 1.5830526 | 0.0 |  |  |  |
|  |  |  |  |  |  |
| **Genes upregulated in sporadic serous cancers** | | | |  |  |
| *Gene symbol* | Fold change | q-value (%) | *Gene symbol* | Fold change | q-value (%) |
| *GPX3* | 0.78854483 | 0.0 | *CLN5* | 0.55048674 | 0.0 |
| *VEZF1* | 0.7056116 | 0.0 | *C1S* | 0.53468794 | 0.0 |
| *AIP* | 0.69674855 | 0.0 | *LRRC32* | 0.501093 | 0.0 |
| *TSPAN9* | 0.67567295 | 0.0 | *RGL1* | 0.480656 | 0.0 |
| *MYL9* | 0.605998 | 0.0 | *PDGFRL* | 0.46526566 | 0.0 |
| *CUTA* | 0.5715599 | 0.0 | *ITGB5* | 0.45047235 | 0.0 |
| *ARHGEF3* | 0.5624485 | 0.0 | *SMARCD3* | 0.43071157 | 0.0 |
|  |  |  |  |  |  |
| *No significant genes identified in the clear cell subgroup | | | |  |  |
